# High-performance symmetric supercapacitors based on carbon nanotube/graphite nanofiber nanocomposites

**DOI:** 10.1038/s41598-018-27460-8

**Published:** 2018-06-13

**Authors:** Yongsheng Zhou, Pan Jin, Yatong Zhou, Yingchun Zhu

**Affiliations:** 1 College of Chemistry and Materials Engineering, Anhui Science and Technology University, Bengbu, 233100 P. R. China; 2 Key Laboratory of Inorganic Coating Materials CAS, Shanghai Institute of Ceramics, Chinese Academy of Sciences, Shanghai, 200050 P. R. China

**Keywords:** Supercapacitors, Structural properties

## Abstract

This work reports the nanocomposites of graphitic nanofibers (GNFs) and carbon nanotubes (CNTs) as the electrode material for supercapacitors. The hybrid CNTs/GNFs was prepared via a synthesis route that involved catalytic chemical vapor deposition (CVD) method. The structure and morphology of CNTs/GNFs can be precisely controlled by adjusting the flow rates of reactant gases. The nest shape entanglement of CNTs and GNFs which could not only have high conductivity to facilitate ion transmission, but could also increase surface area for more electrolyte ions access. When assembled in a symmetric two-electrode system, the CNTs/GNFs-based supercapacitor showed a very good cycling stability of 96% after 10 000 charge/discharge cycles. Moreover, CNTs/GNFs-based symmetric device can deliver a maximum specific energy of 72.2 Wh kg^−1^ at a power density of 686.0 W kg^−1^. The high performance of the hybrid performance can be attributed to the wheat like GNFs which provide sufficient accessible sites for charge storage, and the CNTs skeleton which provide channels for charge transport.

## Introduction

With the ever rising need for environmental friendly, sustainable and high-efficiency energy devices, supercapacitors have attracted tremendous interest for potential applications in electronic circuits, electric vehicles and powering vehicles due to their superior high power density, rate capability, and long cycle-life^1–6^. According to the energy storage mechanism, supercapacitors can be classified as electrochemical double-layer capacitors (EDLCs) and pseudocapacitors. EDLC stores charge by adsorption of electrolyte ions at the electrode/electrolyte interface for energy storage, while pseudocapacitance derives from fast Faradaic reactions at the surface of the electrode^7,8^. However, due to the demand for even longer storage life, existing supercapacitors still need to be improved in terms of its specific capacitance that determine its capability to store energy. The performance of supercapacitors depends intimately on the structure properties of the electrode materials^9–11^.

In order to utilize the merits of supercapacitors, a hybrid electrode has been widely adopted. Carbon materials can supply a good charge transfer path for moving the generated electrical energy in composite electrodes^12–15^, the nanostructured carbon is one of the core electrode materials for high performance supercapacitors^16–18^. Various nanocarbon-based materials including one-dimensional (1D) carbon nanotubes (CNTs) or graphitic nanofibers (GNFs), two- dimensional (2D) graphene nanosheets, and three-dimensional (3D) nanostructured carbon have attracted extensive attention in supercapacitors^16,18–25^. Among these materials, CNTs has superior electrical conductivity, as well as large specific surface area which can dramatically boost the supercapacitance of the carbon composites^26–29^. Graphitic nanofibers (GNFs) is another very interesting carbon material which has virtually open edges and large interlayer spacing^30^. It has been reported that the herringbone GNFs (HBGNFs) are one kind of promising nanocarbons^31^ for supercapacitors due to their cavities, open tips, and graphite platelets, which may provide more active sites and enhance the utilization rate of active materials in the composite electrodes. However, there are limited researches reported the GNFs based supercapacitors. Recently, Cui *et al*. reported a bamboo-like GNFs which exhibited high specific capacitance and rate capability in EDLCs^32^. It has been reported by Lu *et al*. that an excellent specific capacity of 265 F g^−1^ was obtained by a flexible graphene/multiwalled CNTs film^33^. Zhao *et al*.^34^ reported a hybrid of graphene/single-walled CNTs achieved a specific capacitance of 98.5 F g^−1^. It is accepted to all that constructing hierarchical carbon-based composites can combine the structural advances and may achieve enhanced supercapacitive performance. However, the single component in the composite is usually synthesized separately, followed by being mixed together^35^. This synthesis process is complicated and time-consuming. Therefore, it is still a great challenge to pack the sp^2^ carbon layers into the desired 3D structures which is crucial for the supercapacitor applications of these nanostructured carbon.

**Figure 1:**
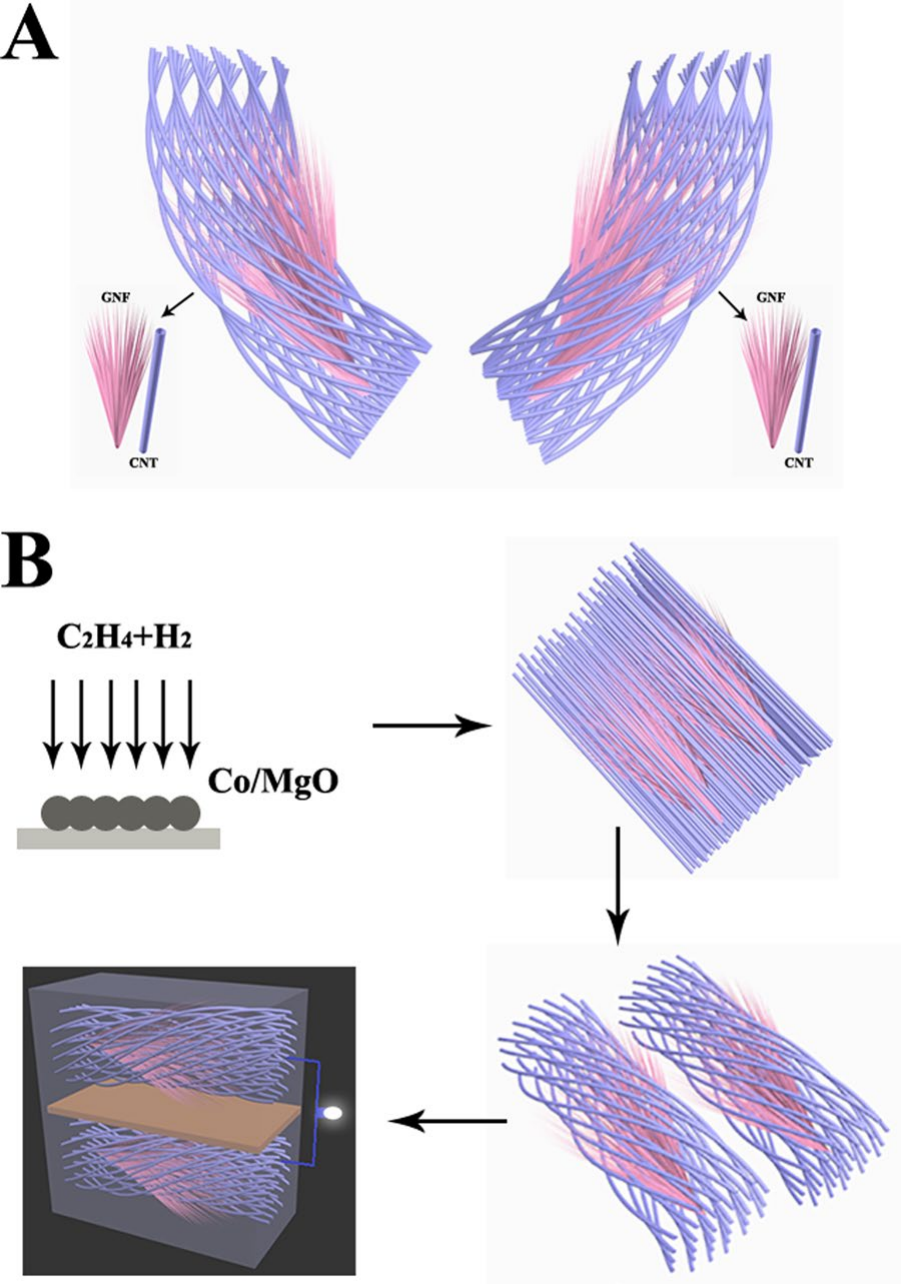
Schematic illustration of the architecture and fabrication of designed CNTs/GNFs symmetric two-electrode. (**A**) Hierarchical CNTs/GNFs structure provide fast channels for charge transport. (**B**) Schematic illustration for the preparation of CNTs/GNFs electrodes.

In this study, we reported a single step synthesis of CNTs/GNFs composites which has never been reported in literature. In the obtained CNTs/GNFs composites, the GNFs has open edges and large interlayer spacing which will ensure ion transportation at high rates and plays the role of an ion reservoir, while the incorporated CNTs are believed to provide greater area for the ions to adhere to and fast channels for charge transport (Fig. 1A). Moreover, the entanglement of CNTs and GNFs can form a mircoporous or a mesoporous network that will further enhance the possible electrostatic adsorption area and channel the ion into the composite active sites. The synthesized CNTs/GNFs composites show a considerable capacitor electrode with a long cycle life and excellent rate capabilities. The method to prepare the CNTs/GNFs hybrid nanocomposites is simple and suitable for mass production. Therefore, a kind of low-cost and environmental friendly electrode materials can be provided for high energy density supercapacitors.

## Results

### Hierarchical CNTs/GNFs

**Figure 2:**
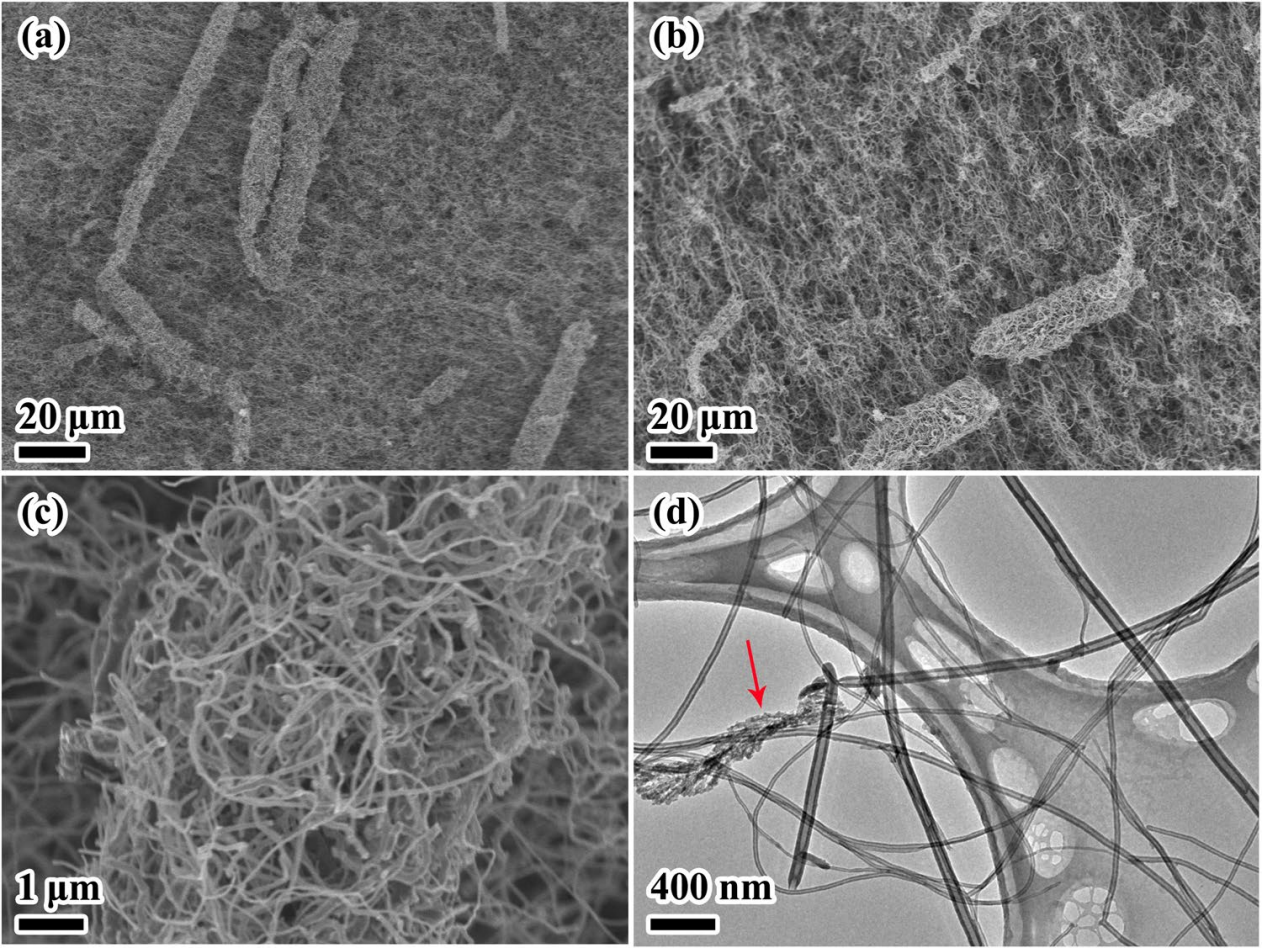
The morphology of the CNTs/GNFs. (**a**,**b**) Low-magnification SEM images; (**c**) High-magnification SEM images. (**d**) TEM images of the CNTs/GNFs composites.

The SEM image of the obtained products shown in Fig. 2 exhibits a long, continuous, and cross-linked carbon network structure. Low magnification SEM images show that CNTs/GNFs composites are intertwined, and present the bird nest type. High magnification SEM image (Fig. 2c) reveals that the nest shaped composites are formed by CNTs and GNFs twisted together. Upon further carefully observation, it can be seen that the GNFs are in a shape like wheat as shown in Fig. 2d (indicated by red arrow). Hybridization of GNF with CNT that illustrates in Fig. 2c and d allows CNT to form a network on the GNF surface. The entanglement of CNTs on the GNFs substrate forms a ‘net’ which captures and promotes ion diffusion into the active sites of the GNFs, thus enhancing the capacitance rate of the CNTs/GNFs hybrid nanocomposites. Apart from FESEM images, TEM images are also obtained in order to study in depth the surface morphology of the samples (Fig. 2d). It is easier to differentiate between CNTs and GNFs via TEM analysis. CNTs display a tube-like structure with a hollow interior similar to a pipeline, while GNFs have a wheat shape with solid interior and many pores which may increase the surface areas of the GNFs. Moreover, the attachment of CNTs promote a greater surface for the electrochemical reaction to occur. Since the mean diameter of GNFs are larger than CNTs, it is convenient for CNTs to attach onto GNFs surfaces.

Figure S-1 (Electronic Supplementary Information) presents the Raman spectrum of the CNTs/GNFs composites. There are two peaks at about 1355 cm^−1^ (D-band), and 1582 cm^−1^ (G-band). The crystallization degree of carbon can be evaluated by the intensity ratio of D peak to G peak (I_D_ /I_G_). The I_D_ /I_G_ of CNTs/GNFs is calculated to be 0.85, implying that the obtained composites mainly comprise the partially graphitic carbon^36–38^. To investigate the structure of the obtained carbon electrode materials, XRD analysis is conducted and the patterns are shown in Fig. S-2 (Electronic Supplementary Information). It can be observed that the CNTs/GNFs composites show two broad diffraction peaks at 43.5 and 25°, these can be assigned to (101) and (002) planes of the materials, respectively, indicating the existence of graphitic carbon. According to the XRD result, we infer that the mass ratio of the GNFs in the sample is about 23%.

To have a better understanding of the textural structures of the CNTs/GNFs materials, we adopt the N_2_ adsorption/desorption isotherm measurements, which results can be seen in Fig. S-3a (Electronic Supplementary Information). It is obvious that lower relative pressures can contribute to the increase of the curves, which indicates the presence of micropores. Apparently, the mesopores can be presented when the hysteresis loops are at medium relative pressures. The BJH (Barrette-Joynere-Halenda) model is employed to calculate the pore size distributions (PSDs). The pore size distribution of the CNTs/GNFs materials is demonstrated in Fig. S-3b (Electronic Supplementary Information). Notably, compared to CNTs, the CNTs/GNFs composite electrode material shows larger pore volume of 0.210 cm^3^ g^−1^ and higherspecific surface area of 1863.1 m^2^ g^−1^. The high specific surface area of the composite material would result in numerous charges can be accommodated during the electrochemical processes.

### Electrochemical performance of CNTs/GNFs in aqueous electrolyte

**Figure 3:**
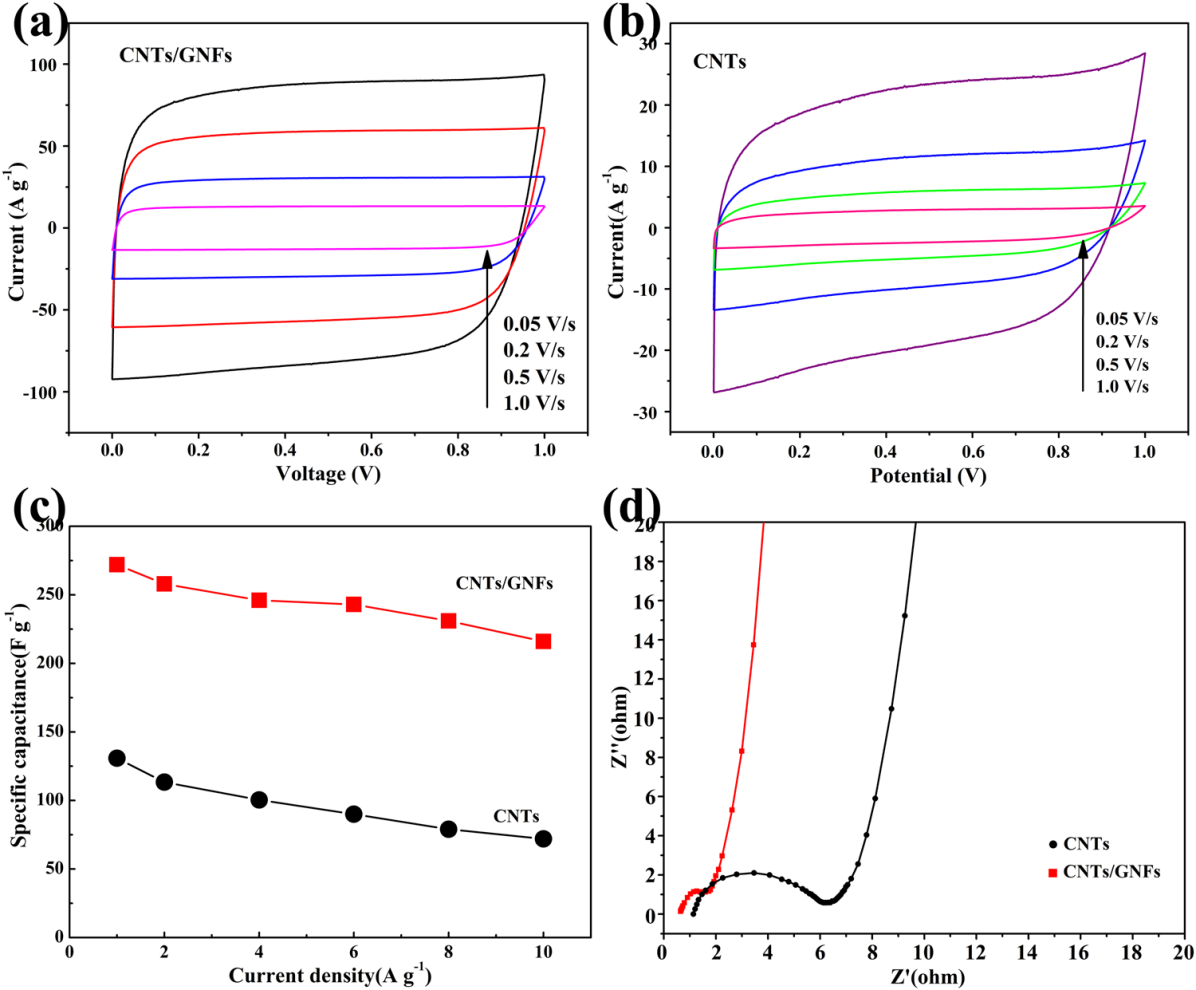
Electrochemical properties of the CNTs/GNFs- and CNTs-based supercapacitros in 6 mol/L KOH electrolyte. CV curves of (**a**) CNT/GNFs and (**b**) CNTs under various scan rates. (**c**) The specific capacitance of CNTs/GNFs and CNTs calculated at various current densities. (**d**) EIS measurements of CNTs/GNFs and CNTs-based EDL supercapacitors.

Next, the CNTs/GNFs were assembled in a three-electrode system to investigate its EDL capacitance in a 6 mol/L KOH electrolyte. For comparison, CNTs without GNFs were also prepared. Figure 3a,b shows the compared CV curves of CNTs/GNFs and CNTs with a scan rate ranging from 0.05 to 1.0 V s^−1^. Both CV curves appear nearly rectangular, indicating a nearly ideal capacitive behavior. The CNTs/GNFs sample displays larger cycle curve area compared to the CNTs sample which displays a relatively small rectangular shape of EDLC. Figure S-4 (Electronic Supplementary Information) and S-5 (Electronic Supplementary Information) show the galvanostatic charge/discharge (GCD) results for samples of CNTs/GNFs and CNTs, respectively. The results indicate that both CNTs and CNTs/GNFs electrodes exhibit a typical symmetric triangular shape for supercapacitors, indicating nearly ideal capacitive behaviors. The specific capacitance of supercapacitors calculated from discharge curve was shown in Fig. 3c. It can be seen that the specific capacitance decreased with the increase of current density. When the current density is 1 A g^−1^, the capacitance is 270 F g^−1^, and 221 F g^−1^ remained when the current density increased to 10 A g^−1^. However, when the current density is 1 A g^−1^, the capacitance of the carbon nanotube is 131 F g^−1^, and when the current density increases to 10 A g^−1^, the capacitor drops to 72 F g^−1^. This can be ascribed to the hierarchical carbon nanostructure of CNTs/GNFs which combine the structural advances of GNF and CNT to improve charge storage. This can be interpreted by the electrochemical impedance spectroscopy (EIS) measurements shown in Fig. 3d. Nyquist plots obtained from EIS show that CNTs/GNFs- and CNTs-based supercapacitors both have a semicircle in the high-frequency region and a straight line in the low-frequence region. The x-intercept at the left end of the semicircle represents the equivalent series resistance (ESR) of the capacitor, which indicates the contact resistance at the active materials and current collector interface, the entire resistances of the ionic resistance of the electrolyte, and the resistance of the electrode material itself. The ESR value of 0.71 ohm for the CNTs/ GNFs-based supercapacitor is smaller than that of CNTs-based supercapacitor (1.09 ohm), which means that CNTs/GNFs-based supercapacitor can be charged/discharged more rapidly. The diameter of the arc attributes to the charge transfer resistance (Rt) at the interface of the electrode and electrolyte. It was obvious that the Rt value for the CNTs/GNFs-based supercapacitor is much lower than that of the CNTs-based supercapacitor. The smaller semicircles diameter of CNTs/GNFs show the low impedance, which is more beneficial for electrolyte ion diffusion and charge transfer. In the low-frequency region, the straight line represents the capacitive performance of ideal materials. The electrochemical performance of the physical mixture of CNT and GNF is also studied (Fig. S-6, Electronic Supplementary Information), however, the capacitance is lower than that of the capacitance of CNTs/GNFs.

### Electrochemical performance of CNTs/GNFs in organic electrolyte

**Figure 4:**
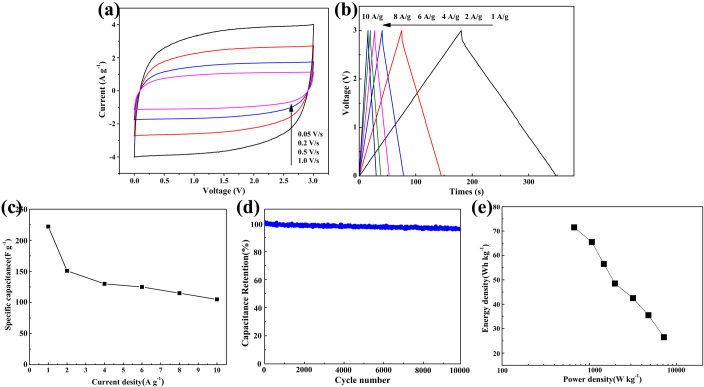
Electrochemical performances of the CNTs/GNFs in an organic electrolyte (NaClO_4_ in EC/DMC). (**a**) CV curves at scan rates from 0.05 to 1 mV s^−1^. (**b**) GCD curves under various current densities. (**c**) The specific capacitance of CNTs/GNFs calculated at various current densities. (**d**) Cycling stability tests at 2 A g^−1^. (**e**) The Ragone plots of supercapacitor.

The energy density of supercapacitor is proportional to the square of voltage. To further explore the practical application of the as-prepared materials for supercapacitors, a serries of electrochemical characterization were performed in an organic electrolyte (NaClO_4_ in EC/DMC) because of its wider electrochemical window. The CV curves of the as-obtained electrode material in symmetric supercapacitors are shown in Fig. 4a. It can be easily proved that the rectangular shaped and nearly symmetric CV curve indicates its excellent capacitive properties. Even at an ultrahigh scan rate of 1 V s^−1^, the CV curves still show quasi-rectangular shapes. Such typical behavior of EDL capacitor suggests the high rate capability and low internal resistance. The galvanostatic charge-discharge (GCD) curves measured at different current densities from 1 to 10 A g^−1^ show good symmetry and nearly linear discharge slopes (Fig. 4b), implying the feature of EDL capacitor as well. The specific capacitance of CNTs/GNFs calculated at various current densities are shown in Fig. 4c. The CNTs/CNFs composites have a specific capacitance of 223 F g^−1^ at a current density of 1 A g^−1^, and decreased to 113 F g^−1^ (10 A g^−1^). It is necessary for energy storage devices with excellent long cycle stability in the practical applications. The cycling stability of the CNTs/GNFs composites electrode at 2 A g^−1^ is shown in Fig. 4d. Even though charging-discharging process exceeds 10 000 cycles, the capacitance remains as high as 96% without any capacitance decay, indicating a superior electrochemical cycling stability.

To further evaluate the applicability of CNTs/GNFs-Cell, Ragone plot was conducted. The energy and power densities of the supercapacitor are calculated from the CNTs/GNFs symmetric supercapacitor in aqueous solution. Obviously, the symmetric supercapacitor displays the maximum energy density of super capacitor with 72.2 Wh kg^−1^ at a power density of 686.0 W kg^−1^, as shown in Fig. 4e, these results are much higher than many previously reported works (Table S1). These results show that CNTs/GNFs composite are promising electrode materials for electrochemical supercapacitors with high performance and excellent rate electrochemical supercapacitor.

## Discussion

In this study, through analyzing the structure and electrochemical properties of electrode materials, we speculated the mechanism of the high performance supercapacitor. The CNTs-based supercapacitor performed poor capacitance due to the randomly bundled CNTs in the electrode, which resulted in disordered transmission pathways and less charge accommodation. In the obtained CNTs/GNFs composites, the GNFs has open edges and large interlayer spacing which will ensure ion transportation at high rates and plays the role of an ion reservoir, while the incorporated CNTs are believed to provide greater area for the ions to adhere to and fast channels for charge transport. Moreover, the entanglement of CNTs and GNFs can form a mircoporous or a mesoporous network that will further enhance the possible electrostatic adsorption area and channel the ion into the composite active sites. Therefore, the excellent performance of the hybrid performance can be attributed to the synergistic behavior of the materials, i.e., wheat like GNFs provide sufficient accessible sites for charge storage and accumulation, while the CNTs backbone provide channels for charge transport.

In summary, a rational experimental route has been successfully formulated for synthesis of a novel 3D hierarchical porous for use as low-cost high-performance electrode materials for supercapacitors. When assembled in a symmetric two-electrode system, the CNTs/GNFs-based supercapacitor showed a very good cycling stability of 96% after 10 000 charge/discharge cycles at 2 A g^−1^. Moreover, the symmetric device based on CNTs/GNFs delivers the maximum energy density of super capacitor with 72.2 Wh kg^−1^ at a power density of 686.0 W kg^−1^. Given its excellent performance, we believe the CNTs/GNFs-based supercapacitor will have great potential for practically wearable and portable electronic applications.

## Method

### Preparation of CNTs/GNFs

The Co/MgO catalyst was prepared as reported^7^. Co(NO_3_)_2_•6H_2_O and Mg(NO_3_)_2_•6H_2_O were mechanically mixed and grounded and then calcined at 600 °C for 1 h in air to decompose the precursor and yield the cluster made of Co and Mg oxides. The resulted powder was then reduced in H_2_ (100 sccm) and Ar (200 sccm) for 30 min at 600 °C to form Co nanoclusters supported on MgO particles, which was collected and used as the catalyst. The synthesis of the CNT/GNF was achieved by the pyrolysis of C_2_H_4_ on Co/MgO catalyst. The Co/MgO catalyst particles were placed in a graphite crucible enclosed within a graphite susceptor using an induction furnace, heated up to the reaction temperature with a flow of C_2_H_4_ (1000 sccm), Ar (800 sccm), and H_2_ (100 sccm). The temperature of the susceptor was controlled to ensure that Co/MgO catalyst particles were heated to 1000 °C. After growth for 15 min, the H_2_ flow was stopped and the chamber was cooled down to room temperature. During the cooling process, the system was purged with Ar to prevent a backflow of air from the exhaust line.

### Preparation of CNTs

The CNTs material was prepared in a way similar to that for the CNTs/GNFs material, but with a flow of C_2_H_4_ (800 sccm), Ar (1700 sccm), and H_2_ (100 sccm).

### Preparation of physical mixture of GNF and CNT

GNF was supplied from Graphene Nanochem Sdn. Bhd. 50 wt% of purified CNT was first added to the GNF sample which was dispersed in 100 ml of deionized water and sonicated for an hour. The mixture was further magnetic stirring for 10 hours. The resultant products were then rinsed with deionized water and dried at 60 °C.

### Characterization

The products were characterized by scanning electron microscopy (SEM), and high-resolution transmission electron microscopy (HRTEM, JEM-2010). X-ray powder diffraction (XRD) pattern of the sample was recorded using a D/max-3C diffractometer equipped with Cu-Kα X-ray source. Raman spectrum was recorded at room temperature with a LABRAMHR Confocal Laser MicroRaman Spectrometer. N_2_ sorption analysis was conducted on an ASAP2020 accelerated surface area and porosimetry instrument (Micromeritics), equipped with automated surface area, at 77 K using BarrettEmmettTeller (BET) calculations for the surface area. The pore size distribution (PSD) plot was recorded from the adsorption branch of the isotherm based on the density functional theory (DFT) model.

### Electrochemical Characterization

The preparation of electrode and analytical measurements of electrochemical properties are described as follows. For the preparation of the activated CNTs/GNFs or CNTs materials, a mixture of KOH and CNTs/GNFs or CNTs (500 wt %) was first heated at 250 °C for 30 min and then treated at 750 °C for 1 h in a tube furnace in Ar gas. The activated mixture was further treated with hot concentrated HNO_3_ to remove the Co/MgO catalyst and obtain the activated CNTs/GNFs or CNTs materials. The resultant products were then rinsed with deionized water and dried in vacuum. Finally, 5 mg of the activated CNTs/GNFs or CNTs materials were pressed into electrode films using a standard preforming mold with a pressure of 5 t. Prototype ECs were assembled using a similar procedure reported in the literature. The aqueous electrolyte based supercapacitor: three-electrode system was carried out where CNTs/GNFs film was used as working electrode, platinum plate was used as the counter electrode (20 mm × 30 mm), Ag/AgCl was employed as the reference electrode, and electrolyte was 6 mol/L KOH solution. The organic electrolyte based supercapacitor: two-electrode system, in which the supercapacitor was constructed by two as-prepared CNTs/GNFs nanocomposite electrodes separated by a porous polypropylene (Celgard 3401, USA) separator and filled with NaClO_4_ in EC/DMC (1:1 vol/vol). The CNTs based supercapacitor was fabricated by the same process. The cyclic voltammetry (CV), galvanostatic charge-discharge (GCD), and Electrochemical Impedance spectroscopy (EIS) measurements were carried out by an electrochemical workstation (CHI 760E). CV curves and GCD at different current densities were performed in the potential window of 0 to 1.0 V. The calculation of specific capacitance GCD curves:

1a $${C}_{sp}=\frac{I\varDelta t}{m\varDelta V}$$

where I (A) is the discharge current, Δt(s) is the discharge time, m(g) is the mass of the single working electrode, and ΔV(V) is the voltage change during the discharge process.

The calculation of specific capacitance by GCD curves in three-electrode configuration:

1b $${C}_{s1}=\frac{I}{(m\bullet (\frac{dV}{dt}))}$$

The calculation of specific capacitance by GCD curves in two-electrode configuration:

1c $${C}_{s2}=\frac{4I}{(m\bullet (\frac{dV}{dt}))}$$

The volumetric energy (E) and power (P) density of the SCs were obtained from the equations:

2 $$E=\frac{1}{2\times 3.6}C{V}^{2}$$

3 $$P=\frac{E}{t}$$

where C is the specific capacitance of the total symmetric system, V is voltage change during discharge process after IR drop in V-t curve and t is the discharge time.

## Electronic supplementary material

Supplementary Information
