# Author Correction: High-performance symmetric supercapacitors based on carbon nanotube/graphite nanofiber nanocomposites

**DOI:** 10.1038/s41598-021-99515-2

**Published:** 2021-10-20

**Authors:** Yongsheng Zhou, Pan Jin, Yatong Zhou, Yingchun Zhu

**Affiliations:** 1 College of Chemistry and Materials Engineering, Anhui Science and Technology University, Bengbu, 233100 P. R. China; 2 Key Laboratory of Inorganic Coating Materials CAS, Shanghai Institute of Ceramics, Chinese Academy of Sciences, Shanghai, 200050 P. R. China

Correction to: *Scientific Reports* 10.1038/s41598-018-27460-8, published online 13 June 2018

The original version of this Article contained errors.

In the Results section, under subheading “Electrochemical performance of CNTs/GNFs in organic electrolyte”,

“The galvanostatic charge-discharge (GCD) curves measured at different current densities from 0.5 to 10 A g^−1^ show good symmetry and nearly linear discharge slopes (Fig. 4b), implying the feature of EDL capacitor as well.”

now reads:

“The galvanostatic charge-discharge (GCD) curves measured at different current densities from 1 to 10 A g^−1^ show good symmetry and nearly linear discharge slopes (Fig. 4b), implying the feature of EDL capacitor as well.”

As a result of the changes, Figure 4b was incorrect. The coloured lines and corresponding arrow labels were incorrectly given.

The original Figure 4 and accompanying legend appear below.

**Figure 4:**
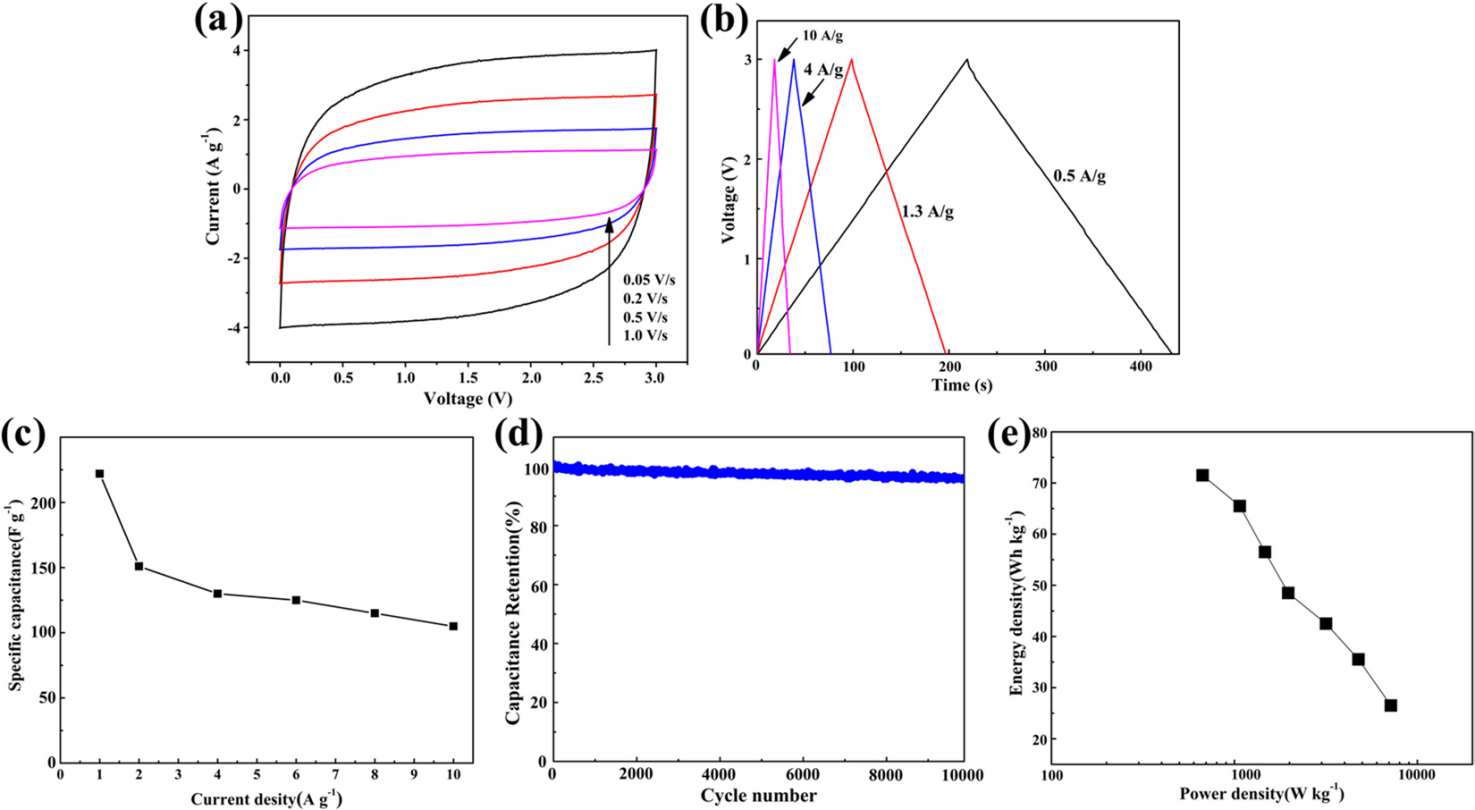
Electrochemical performances of the CNTs/GNFs in an organic electrolyte (NaClO_4_ in EC/DMC). (**a**) CV curves at scan rates from 0.05 to 1 mV s^−1^. (**b**) GCD curves under various current densities. (**c**) The specific capacitance of CNTs/GNFs calculated at various current densities. (**d**) Cycling stability tests at 2 A g^−1^. (**e**) The Ragone plots of supercapacitor.

Lastly, the explanation provided for Equation 1 was incomplete and now includes parts 1b and 1c.

“The calculation of specific capacitance GCD curves:

1 $${C}_{sp}=\frac{I\Delta t}{m\Delta V}$$

where I (A) is the discharge current, Δt(s) is the discharge time, m(g) is the mass of the single working electrode, and ΔV(V) is the voltage change during the discharge process.”

now reads:

“The calculation of specific capacitance GCD curves:

1a $${C}_{sp}=\frac{I\Delta t}{m\Delta V}$$

where I (A) is the discharge current, Δt(s) is the discharge time, m(g) is the mass of the single working electrode, and ΔV(V) is the voltage change during the discharge process.

The calculation of specific capacitance by GCD curves in three-electrode configuration:

1b $${C}_{s1}=\frac{I}{(m\bullet (\frac{dV}{dt}))}$$

The calculation of specific capacitance by GCD curves in two-electrode configuration:

1c $${C}_{s2}=\frac{4I}{(m\bullet (\frac{dV}{dt}))}$$

The original Article has been corrected.

