# Supplementary material for: High-performance symmetric supercapacitors based on carbon nanotube/graphite nanofiber nanocomposites
Source: Sci Rep. 2018 Jun 13;8:9005. doi: 10.1038/s41598-018-27460-8 (PMC5998012; doi:10.1038/s41598-018-27460-8)
Supplement: Supplementary file 1 — Supplementary Information [file 41598_2018_27460_MOESM1_ESM.docx]

High-performance symmetric supercapacitors based on carbon nanotube/graphite nanofiber nanocomposites

*Yongsheng Zhou ^1,2,*^, Pan Jin ^1^, Yatong Zhou ^1^, and Yingchun Zhu ^2^*

1 College of Chemistry and Materials Engineering, Anhui Science and Technology University, Bengbu, 233100, China

2 Key Laboratory of Inorganic Coating Materials, Shanghai Institute of Ceramics, Chinese Academy of Sciences, Shanghai, 200050, China

 E-mail: yszhou1981@gmail.com





Figure S-1 Raman spectra of the CNTs/GNFs.





Figure S-2 XRD pattern of the CNTs/GNFs.


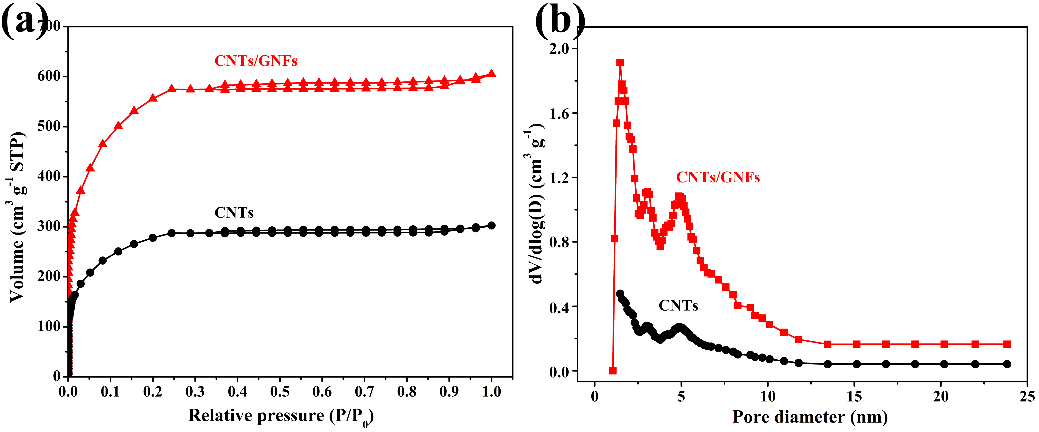


Figure S-3 (a)N_2_ sorption isotherms of CNTs/GNFs and CNTs, and (b)pore size distribution from the BJH method of corresponding samples.





Figure S-4 Galvanostatic charge/discharge curves of CNTs/GNFs at different current densities.





Figure S-5 Galvanostatic charge/discharge curves of CNTs at different current densities.


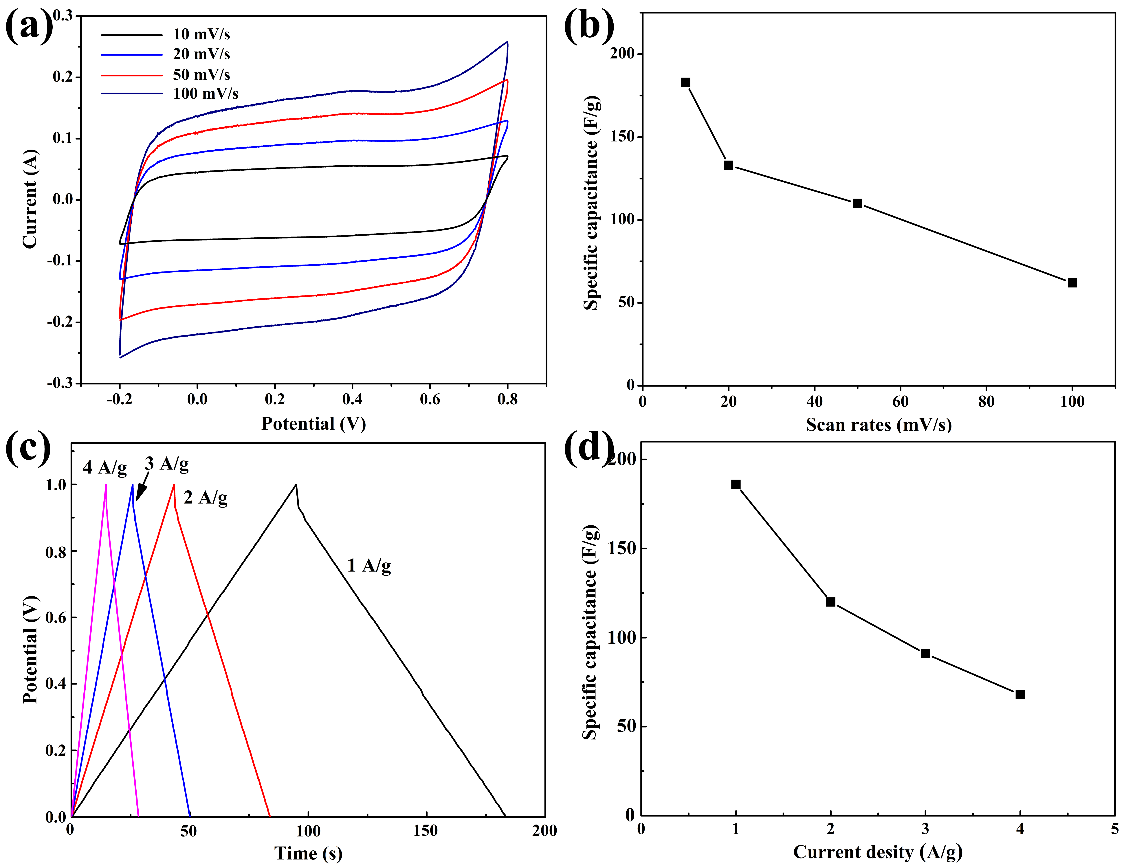


Figure S-6 Electrochemical performance of the physical mixture of CNT and GNF: (a) CV curves at various scan rates, (b) specific capacitance value at different scan rates; (c) Galvanostatic charge/discharge curves at different current densities, (d) specific capacitance value at different current densities.

**Table S1**| Specific capacitance and energy density values of different CNT-based materials for supercapacitors.

| Materials | Capacitance | Energy density | References |
| --- | --- | --- | --- |
| CNT/graphene | 120 F g^-1^ in 1.0 M H_2_SO_4_ |  | 1 |
| Activated carbon wrapped CNT buckypaper | 100 F g^-1^ in 6 M KOH |  | 2 |
| CNT/PEDOT | 179 F cm ^-3^ in H_3_PO_4_/PVA | 1.4 mWh cm ^-1^ | 3 |
| CNT/PPY | 184 F g^-1^ in KCl solution |  | 4 |
| Macro-/meso-porous CNT sponge | 150 F g^-1^ in 6 M KOH |  | 5 |
| CNT supported graphene aerogel | 169.3 F g^-1^ in 6 M KOH |  | 6 |
| VACNTs/CNFs | 213 F g ^-1^ in NaOH | 70.7 Wh kg ^-1^ | 7 |
| CNTs/PANI hydrogel | 315 F g ^-1^ in H_3_PO_4_/PVA |  | 8 |
| GO-OLC-MWCNT | 203.5 F g ^-1^ in 3 M KCl | 45.4 Wh kg ^-1^ | 9 |
| CNT@micro-carbon | 209 F g^-1^ in 6 M KOH |  | 10 |
| Graphene/ CNTs/PANI | 259 F g^-1^ in 5 M KOH |  | 11 |
| Cellulose-Mediated PEDOT:PSS/MWCNT | 50.4 F cm ^-3^ in PVA/KOH |  | 12 |
| Our work | 270 F g ^-1^ in 6 M KOH | 72.2 Wh kg ^-1^ |  |

**References**

1. Yu D. S.; Dai L. M. Self-assembled graphene/carbon nanotube hybrid films for supercapacitors. *J. Phys. Chem. Lett*. **2010**, 1: 467-470.

2. Chen H. Y.; Di J. T.; Jin Y.; Chen M. H.; Tian J.; Li Q. W. Active carbon wrapped carbon nanotube buckypaper for the electrode of electrochemical supercapacitors. *J. Power Sources* **2013**, 237: 325-331.

3. Lee J. A.; Shin M. K.; Kim S. H.; Cho H. U.; Spinks G. M.; Wallace G. G.; Lima M. D.; Lepro X.; Kozlov M. E.; Baughman R. H.; Kim S. J. Ultrafast charge and discharge biscrolled yarn supercapacitors for textiles and microdevices. *Nat. Commun*. **2013**, 4: 2970.

4. Liu F. F.; Han G. Y.; Chang W. Z.; Fu D. Y.; Li Y. P.; Li M.Y. Fabrication of carbon nanotubes/polypyrrole/carbon nanotubes/melamine foam for supercapacitor. *J. Appl. Polym. Sci*. **2014**,131: 39779.

5. Yang Y. B.; Li P. X.; Wu S. T.; Li X. Y.; Shi E. Z.; Shen Q. C.; Wu D. H.; Xu W. J.; Cao A. Y.; Yuan Q. Hierarchically designed three-dimensional macro/mesoporous carbon frameworks for advanced electrochemical capacitance storage. *Chem.—Eur. J*. **2015**, 21: 6157-6164.

6. Ma Z. H.; Zhao X. W.; Gong C. H.; Zhang J. W.; Zhang J. W.; Gu X. F.; Tong L.; Zhou J. F.; Zhang Z. J. Preparation of a graphene-based composite aerogel and the effects of carbon nanotubes on preserving the porous structure of the aerogel and improving its capacitor performance. *J. Mater. Chem. A* **2015**, 3: 13445-13452.

7. Qiu Y. C.; Li G. Z.; Hou Y.; Pan Z. H.; Li H. F.; Li W. F.; Liu M. N.; Ye F. M.; Yang X.W.; Zhang Y. G. Vertically aligned carbon nanotubes on carbon nanofibers: a hierarchical three-dimensional carbon nanostructure for high-energy flexible supercapacitors. *Chem. Mater*. **2015**, 27: 1194-1200.

8. Xiang X.; Zhang W. J.; Yang Z. P.; Zhang Y. Y.; Zhang H. J.; Zhang H.; Guo H. T.; Zhang X. T.; Li Q. W. Smart and flexible supercapacitor based on a porous carbon nanotube film and polyaniline hydrogel. *RSC Advances* **2016**, 6: 24946-24951.

9. Wang C. D.; Liu D. B.; Chen S. M.; Sang Y.; Haleem Y. A.; Wu C. Q.; Xu W. Y.; Fang Q.; Habib M.; Cao J.; Niu Z. Q.; Ajayan P. M.; Song L. All-Carbon Ultrafast Supercapacitor by Integrating Multidimensional Nanocarbons. *Small* **2016**, 12, 5684-5691.

10. Li Z.; Li Z.; Li L.; Li C.; Zhong W.; Zhang H. Construction of hierarchically one-dimensional core-shell CNT@microporous carbon by covalent bond-induced surface-confined crosslinking for high-performance supercapacitor. *ACS Appl. Mater. Interfaces* **2017**, 9 (18): 15557-15565.

11. Jo E.H.; Jang H. J.; Chang H.; Kim S. K.; Choi J.; Lee C. M. High electrochemical performance of three-dimensional network structured crumpled graphene/carbon nanotube/polyaniline composites for supercapacitors. *ChemSusChem* DOI: 10.1002/cssc.201700212.

12. Zhao D. W.; Zhang Q.; Chen W. S.; Yi X.; Liu S. X.; Wang Q. W.; Liu Y. X.; Li J.; Li X. F.; Yu H. P. Highly flexible and conductive cellulose-mediated PEDOT:PSS/MWCNT composite films for supercapacitor electrodes. *ACS Appl. Mater. Interfaces* **2017**, 9 (15): 13213-13222.
